# How We Treat Wounds To-day

**Published:** 1887-01

**Authors:** 


**How we Treat Wounds To-Day.** A Treatise on the Subject of Antiseptic Surgery, which can be understood by beginners. By ROBERT T. MORSE, M. D., late House-Surgeon to Bellevue Hospital, N. Y.; Member Lumæan Society of Natural History, N. Y.; Member New York County Medical Society, Second edition. New York: G. P. Putnam's Son's. 1886.

This work has passed rapidly to a second edition; this alone giving evidence of its popularity and the value the profession places upon it. It contains all that a busy practitioner needs for the complete understanding of Listerism in its minutest detail. It gives a concrete description of the way in which a few wounds should be treated; it then gives facts concerning the methods. After giving pages of good advice on the materials needed and how to prepare them, it gives the places where such can be bought, the price, etc. It then proceeds with the general directions, the treatment of recent incised, inflamed incised, wounds in ovariotomy, wounds remaining exposed, wounds requiring frequent change of dressing, lacerated and contused wounds, gun shot wounds and burns. Lastly he gives words of advice as follows: "He who has not learned some scientific antiseptic method for wound treatment has not learned the first principles of surgery." In which we heartily concur.

